# Predictors of hazardous drinking, tobacco smoking and physical inactivity in vocational school students

**DOI:** 10.1186/1471-2458-13-475

**Published:** 2013-05-15

**Authors:** Severin Haug, Michael P Schaub, Corina Salis Gross, Ulrich John, Christian Meyer

**Affiliations:** 1 Swiss Research Institute for Public Health and Addiction at Zurich University, Konradstrasse 32, 8031, Zurich, Switzerland; 2 University of Greifswald, Institute of Epidemiology and Social Medicine, Walther-Rathenau-Str. 48, 17487, Greifswald, Germany

**Keywords:** Health behaviour, Health risk factors, Adolescents, Young adults, Students

## Abstract

**Background:**

Tobacco smoking, hazardous drinking and physical inactivity during adolescence are risk factors that are associated with poorer health in adulthood. The identification of subgroups of young people with a high prevalence of one or more of these risk factors allows an optimised allocation of preventive measures. This study aimed at investigating hazardous drinking, tobacco smoking and physical inactivity as well as their associations and demographic predictors in vocational school students.

**Methods:**

Out of 57 contacted vocational schools in Switzerland, a total of 24 schools participated in a survey assessing gender, age, immigrant background, educational attainment and vocational field as well as the above mentioned health risk factors. Out of the 2659 students present in 177 included vocational school classes, 2647 (99.5%) completed the survey. Binary logistic regression analyses were conducted to investigate the demographic predictors of each health risk factor and a multinomial logistic regression analysis was conducted to investigate predictors of different risk factor combinations.

**Results:**

Of the surveyed students, 79.4% showed at least one risk factor, 43.6% showed two or more and 9.6% showed all three health risk factors. Hazardous drinking was more prevalent in male, physical inactivity was more prevalent in female vocational school students. The proportion of students with low physical activity and tobacco smoking increased with increasing age. While the combination of hazardous drinking and tobacco smoking was higher in males, the other risk factor combinations were observed particularly among females.

**Conclusions:**

Multiple risk factors were ascertained in a significant proportion of vocational school students. Specifically, tobacco smoking and hazardous drinking were coexistent. The study underlines the need for preventive measures in specific subpopulations of adolescents and young adults with lower educational level.

## Background

Tobacco and alcohol use as well as physical inactivity are major causes of the disease burden in high-income countries [1]. Use of substances and physical inactivity during adolescence are associated with poorer health in adulthood. Early initiation of substance use is associated with an elevated risk of developing substance use disorders [2]. A low level of physical activity during adolescence and young adulthood is associated with a higher risk of cardio-vascular disease in adulthood [3]. Furthermore, a low level of vigorous physical activity is associated with overweight [4]. Therefore, teenagers and adolescents are a major target group for preventive measures to modify these health risk behaviours.

International school surveys conducted in secondary schools, such as the Health Behaviour in School Age Children (HBSC; [5]) or the European School Survey Project on Alcohol and Other Drugs (ESPAD; [6]), provide data regarding health behaviour and the prevalence of health risk factors for children between the ages of 11 and 16 years. Furthermore, numerous studies have addressed the health behaviour of university students [7,8]. However, little is known about health risk behaviours in adolescents and young adults between the ages of 16 and 20 years with a broader spectrum of educational levels. Vocational school students are typically characterised by heterogeneous educational levels, including a significant proportion with little or no educational attainment.

Based on data of the Swiss Federal Statistical Office, approximately half of adolescents aged 16 to 19 years currently attend vocational schools [9], with the highest proportions among adolescents aged 17 (males: 63%, females: 48%) and 18 (males: 63%, females: 49%) years. This occurrence is common in European countries such as Switzerland or Germany.

In a recent study [10], tobacco smoking and alcohol consumption were examined among 1124 vocational school students in a defined area of Northern Germany. Compared to general population survey data for this region, this study revealed higher levels of tobacco use and hazardous drinking with a smoking prevalence of 61% and binge drinking reported in 79% of vocational school students.

To date, few studies have been able to consider the co-occurrence and association of different health risk behaviours in adolescents [4,11-13]. In adolescents from the United States, [11] did not find differences between smokers and non-smokers for moderate and vigorous physical activity. In most countries which participated in the HBSC study, tobacco use was negatively related to physical activity [12].

The aim of this study was to investigate hazardous drinking (also called “alcohol misuse”, which includes the spectrum from drinking above recommended limits to severe alcohol dependence [14]), tobacco smoking and physical inactivity, as well as their associations and demographic predictors in a sample of vocational students mainly aged 16 to 20 years. Based on the results of this study, conclusions are drawn regarding the need for preventive measures in subpopulations of vocational school students. The results concerning the co-occurrence of different health risk behaviours might help to inform future interventions, such as coordinated school health programs.

## Methods

The data for this study were collected within the randomised controlled trial “Efficacy of a text messaging (SMS)-based smoking cessation intervention for adolescents and young adults” (Trial registration: Current Controlled Trials ISRCTN19739792). The study was approved by the Local Ethics Committee of the Canton of Zurich, Switzerland and the Cantonal Office for Secondary Education in Zurich.

### Sample recruitment

In Switzerland, vocational schools are typically post-secondary public schools and are analogous to American community colleges. They are part of the dual educational system that combines apprenticeships in a business-context and vocational training in a school-context. Vocational schools provide general education (including sports lessons) and specific skills for each particular profession.

Directors of vocational schools and contact teachers for addiction prevention from 57 vocational schools in the Swiss cantons of Zurich, Aargau, Basel, Zug and Schwyz were invited to participate with some of their classes in a study testing the efficacy of a text messaging-based smoking cessation program. Of these schools, 24 vocational schools with a total of 177 school classes agreed to participate in the study. All students of the participating vocational school classes were invited by externally trained staff to participate in an anonymous online health survey during a regular school lesson reserved for health education. Participation was on a voluntary basis. To decrease reporting bias, the study assistants did not provide further information about the purpose of the study before the anonymous screening assessment was completed. The assessments were conducted between October 2011 and May 2012. At the time of the assessment, 2659 students were present in the school classes, of whom 2647 (99.5%) completed the assessment.

### Instruments

The screening assessment included demographics, alcohol consumption, smoking status and physical activity. We assessed the following demographic variables: gender, age, school education, immigrant background and vocational field. Common Swiss levels of educational attainment were assessed: (a) none, (b) secondary school, (c) extended secondary school and (d) technical or high school. We assessed the country of birth of both parents of the students to identify a potential immigrant background. For the analyses, we collapsed (a) persons with neither parent born outside Switzerland, (b) persons with one parent born outside Switzerland and (c) persons with both parents born outside Switzerland. The vocational fields were classified according to a list from the Swiss Service Centre for Occupational Training [15], in which all jobs that require training are assigned to one of 22 vocational fields. An additional category was created for persons attending vocational preparation.

Alcohol consumption was assessed by the first three items of the Alcohol Use Disorder Identification Test (AUDIT, [16]), the AUDIT-C [17]. The AUDIT–C assesses drinking quantity, drinking frequency and binge drinking and considers drinking behaviour of the previous year. Based on recent recommendations [14], we used the gender-specific cut-off values, i.e., ≥ 4 for men and ≥ 3 for women, to determine whether hazardous or risky drinking was present.

Tobacco smoking was assessed by the question, “Are you currently smoking cigarettes or did you smoke in the past?” with the following response options: (a) I smoke cigarettes daily; (b) I smoke cigarettes occasionally, but not daily; (c) I smoked cigarettes in the past, but I do not smoke anymore; and (d) I have never smoked cigarettes or have smoked less than 100 cigarettes in my life. Current daily and occasional smokers (categories a and b) were considered smokers.

Self-reported moderate to vigorous physical activity (VPA) was measured by a question derived from the HBSC study [18]: “Outside school: How many hours a week do you exercise or participate in sports that make you sweat or out of breath?”. Individuals who reported < 2 hours of extracurricular VPA per week were considered physically inactive. This cut-off is based on the recommendations of the World Health Organization [19] that adults should be moderately physically active for at least 150 minutes per week. As only whole numbers without decimal places could be entered by the participants and education in vocational schools typically includes physical education for at least 45 minutes per week, we used a cut-off point of less than two hours of extracurricular VPA per week.

### Data analysis

We initially calculated correlation coefficients between the investigated risk factors, hazardous drinking, tobacco smoking and physical inactivity, using the phi coefficient. Subsequently, separate binary logistic regression analyses were conducted to explore the following demographic predictors of each health risk factor: gender, age, educational attainment, immigrant background and vocational field. The reference group within these binary logistic regression models were persons without the specific examined risk factor. To obtain comparable results for the different risk factors, we entered all demographic variables as covariates into each regression model and did not conduct variable selection methods. To predict the prevalence of the 4 different risk factor combinations, we conducted a multinomial logistic regression model. We used persons with no or exactly one risk factor as the reference group of the dependent variable; the risk factor combinations (1) drinking and smoking, (2) drinking and inactivity, (3) smoking and inactivity and (4) drinking and smoking and inactivity constituted further categories of the dependent variable. Due to a low number of persons in single categories of the variable “vocational field” this variable could not be included as predictor within the multinomial logistic regression model. Given the clustered nature of the data (students within school classes) we computed robust variance estimators for all logistic regression models. All analyses were performed using STATA, version 10. An alpha level of .05 (2-tailed) was chosen for the statistical tests.

## Results

### Sample characteristics

Demographic characteristics of the study sample are depicted in Table 1; health related characteristics are displayed in Table 2.

**Table 1:** Demographic characteristics of the study sample

**All subjects**	**2647**
Female Gender	1346 (50.9%)
Age, *Md* (*Q1; Q3*)	18.00 (17.00; 19.00)
15-16 years	465 (17.6%)
17-18 years	1351 (51.0%)
19-20 years	622 (23.5%)
21 years or older	209 (7.9%)
Educational attainment	
None	69 (2.6%)
Secondary school (9 years)	2086 (78.8%)
Extended secondary school (10 or 11 years)	383 (14.5%)
Technical or high school	109 (4.1%)
Immigrant background	
No immigrant background	1424 (53.8%)
One parent born outside Switzerland	461 (17.4%)
Both parents born outside Switzerland	762 (28.8%)
Vocational field	
Nature (agriculturist, gardener, forester)	172 (6.5%)
Gastronomy, food (baker, waiter)	28 (1.1%)
Beauty, sports (hairdresser)	62 (2.3%)
Design, art, print (graphic designer, screen printer, photographer)	84 (3.2%)
Construction and interior fitting (bricklayer, painter, carpenter)	87 (3.3%)
Electrical engineering, information technology	316 (11.9%)
Metal and machinery (mechanic, plant engineer)	121 (4.6%)
Chemistry, physics (laboratory worker, lacquerer)	28 (1.1%)
Planning and construction (draughtsman, modeler)	100 (3.8%)
Sale (shopkeeper, retailer)	106 (4.0%)
Economy and administration (commercial employee)	627 (23.7%)
Traffic, logistics, vehicles (logistician)	220 (8.3%)
Health (health profession, dental assistant)	532 (20.1%)
Education and social affairs (kindergarten teacher, care provider)	78 (2.9%)
Vocational preparation	86 (3.2%)

Values are numbers unless stated otherwise.

**Table 2:** Health related characteristics of the study sample

**All subjects**	**2647**
Alcohol consumption, AUDIT-C (scale 0–12),	
Males, *Md* (*Q1; Q3*)	5.00 (3.00; 7.00)
Females, *Md* (*Q1; Q3*)	3.00 (1.00; 5.00)
AUDIT-C < 4 in males	413 (31.7%)
AUDIT-C ≥ 4 in males	888 (68.3%)
AUDIT-C < 3 in females	586 (43.5%)
AUDIT-C ≥ 3 in females	760 (56.5%)
Tobacco smoking	
Current daily smokers	778 (29.4%)
Current occasional smokers	330 (12.5%)
Recent quitters	215 (8.1%)
Never smokers	1324 (50.0%)
Hours of extracurricular moderate to vigorous physical activity per week,	
*Md* (*Q1; Q3*)	3.00 (1.00; 5.00)
0	376 (14.2%)
1	365 (13.8%)
2 – 3	759 (28.7%)
4 – 5	500 (18.9%)
6 – 7	291 (11.0%)
8 – 9	133 (5.0%)
10 or more	223 (8.4%)

Values are numbers unless stated otherwise.

### Health risk factors

According to the AUDIT-C, the prevalence of hazardous drinking was 62.2% in the total sample, with 68.3% in males and 56.5% in females. Thirty-seven per cent (36.9%) of the total sample were current daily or occasional smokers, and 28.0% reported less than two hours of extracurricular moderate to vigorous physical activity per week.

Among all participants, 20.6% (n = 545) had none of the three health risk factors investigated, 35.9% (n = 950) had one (hazardous drinking: 21.8%, n = 578; tobacco smoking: 4.7%, n = 125; physical inactivity: 9.3%, n = 247) 34.0% (n = 899) had two (hazardous drinking & tobacco smoking: 24.5%, n = 648; hazardous drinking and physical inactivity: 6.4%, n = 169; tobacco smoking and physical inactivity: 3.1%, n = 82), and 9.6% (n = 253) had all three health risk factors.

We found a low inverse correlation between hazardous drinking and physical inactivity (*Φ* = −.08, *p* < .01) and a moderate positive association between hazardous drinking and tobacco smoking (*Φ* = .33, *p* < .01), while tobacco smoking and physical inactivity were not associated (*Φ* = .04, *p* = .07).

### Predictors of hazardous drinking

The multiple prediction model of hazardous drinking (Table 3) showed significantly lower prevalence of hazardous drinking in females (OR = 0.71, 95% CI = 0.56-0.89, *p* < .01) compared to males, in persons with both parents born outside Switzerland (OR = 0.34, 95% CI = 0.28-0.42, *p* < .01) compared to persons with both parents born in Switzerland as well as in persons in vocational preparation (OR = 0.42, 95% CI = 0.23-0.75, *p* < .01) compared to persons from the vocational field “nature”, which was defined as the reference group.

**Table 3:** Multiple prediction model of risk factor “hazardous drinking” (reference group: vocational school students without this risk factor)

**Variable**	**OR (95% CI)**
Gender: Male (ref.)	
Female	0.71 (0.56-0.89)**
Age: 15-16 years (ref.)	
17-18 years	1.28 (0.99-1.67)
19-20 years	1.29 (0.93-1.79)
21 years or older	1.15 (0.75-1.78)
Educational attainment: None (ref.)	
Secondary school	0.70 (0.41-1.21)
Extended secondary school	0.68 (0.37-1.24)
Technical or high school	0.94 (0.45-1.95)
Immigrant background: No (ref.)	
One parent born outside Switzerland	0.97 (0.76-1.23)
Both parents born outside Switzerland	0.34 (0.28-0.42)**
Vocational field: Nature (ref.)	
Gastronomy, food	1.01 (0.51-1.99)
Beauty, sports	1.21 (0.72-2.05)
Design, art, print	1.66 (0.97-2.83)
Construction and interior fitting	1.09 (0.58-2.07)
Electrical engineering, information technology	1.49 (0.91-2.44)
Metal and machinery	1.05 (0.53-2.08)
Chemistry, physics	0.75 (0.40-1.39)
Planning and construction	1.10 (0.73-1.66)
Sale	1.06 (0.68-1.64)
Economy and administration	1.09 (0.70-1.69)
Traffic, logistics, vehicles	1.07 (0.62-1.83)
Health	0.80 (0.51-1.26)
Education and social affairs	1.46 (0.79-2.67)
Vocational preparation	0.42 (0.23-0.75)**

*Note:* *p < .05, **p < .01. OR = Odds Ratio; CI = Confidence Interval.

### Predictors of tobacco smoking

The prediction model for tobacco smoking (Table 4) revealed a higher smoking prevalence in persons in the older age groups (19–20 years: OR = 1.39, 95% CI = 1.06-1.83, *p* < .05; 21 years or older: OR = 1.52, 95% CI = 1.07-2.15, *p* < .05) compared to the reference age group of 15–16 years, in persons with one parent born outside Switzerland (OR = 1.31, 95% CI = 1.04-1.65, *p* < .05) compared to those with both parents born in Switzerland and in persons from the “gastronomy, food” (OR = 2.55, 95% CI = 1.29-5.06, *p* < .01) and “beauty, sports” (OR = 2.13, 95% CI = 1.06-4.26, *p* < .05) vocational fields compared to persons from the “nature” vocational field.

**Table 4:** Multiple prediction model of risk factor “tobacco smoking” (reference group: vocational school students without this risk factor)

**Variable**	**OR (95% CI)**
Gender: Male (ref.)	
Female	0.93 (0.75-1.14)
Age: 15-16 years (ref.)	
17-18 years	1.18 (0.93-1.50)
19-20 years	1.39 (1.06-1.83)*
21 years or older	1.52 (1.07-2.15)*
Educational attainment: None (ref.)	
Secondary school	0.78 (0.47-1.29)
Extended secondary school	0.80 (0.46-1.39)
Technical or high school	0.79 (0.44-1.42)
Immigrant background: No (ref.)	
One parent born outside Switzerland	1.31 (1.04-1.65)*
Both parents born outside Switzerland	0.95 (0.77-1.17)
Vocational field: Nature (ref.)	
Gastronomy, food	2.55 (1.29-5.06)**
Beauty, sports	2.13 (1.06-4.26)*
Design, art, print	1.16 (0.68-1.98)
Construction and interior fitting	0.69 (0.33-1.44)
Electrical engineering, information technology	1.21 (0.80-1.84)
Metal and machinery	1.02 (0.56-1.85)
Chemistry, physics	1.93 (0.60-6.13)
Planning and construction	0.99 (0.63-1.55)
Sale	1.63 (0.91-2.92)
Economy and administration	0.85 (0.55-1.32)
Traffic, logistics, vehicles	1.22 (0.78-1.91)
Health	1.12 (0.70-1.80)
Education and social affairs	1.76 (0.79-3.90)
Vocational preparation	1.17 (0.60-2.30)

*Note:* *p < .05, **p < .01. OR = Odds Ratio; CI = Confidence Interval.

### Predictors of physical inactivity

Significantly higher prevalence of physical inactivity (Table 5) were identified in females compared to males (OR = 2.65, 95% CI = 2.08-3.39, *p* < .01). Compared to the youngest age group, physical inactivity was more prevalent in older vocational school students (17–18 years: OR = 1.34, 95% CI = 1.01-1.78, *p* < .05; 19–20 years: OR = 1.37, 95% CI = 1.00-1.88, *p* < .05; 21 years or older: OR = 1.95, 95% CI = 1.25-3.05, *p* < .01). Compared to persons from the reference vocational field, “nature”, we found lower prevalence of physical inactivity in those from the “construction and interior fitting” vocational field (OR = 0.42, 95% CI = 0.19-0.94, *p* < .05) and higher prevalence among those in vocational preparation (OR = 1.75, 95% CI = 1.00-3.04, *p* < .05).

**Table 5:** Multiple prediction model of risk factor “physical inactivity” (reference group: vocational school students without this risk factor)

**Variable**	**OR (95% CI)**
Gender: Male (ref.)	
Female	2.65 (2.08-3.39)**
Age: 15-16 years (ref.)	
17-18 years	1.34 (1.01-1.78)*
19-20 years	1.37 (1.00-1.88)*
21 years or older	1.95 (1.25-3.05)**
Educational attainment: None (ref.)	
Secondary school	1.05 (0.62-1.76)
Extended secondary school	1.11 (0.62-1.98)
Technical or high school	1.18 (0.58-2.44)
Immigrant background: No (ref.)	
One parent born outside Switzerland	1.13 (0.87-1.46)
Both parents born outside Switzerland	1.20 (0.97-1.49)
Vocational field: Nature (ref.)	
Gastronomy, food	0.95 (0.62-1.46)
Beauty, sports	1.84 (0.65- 5.21)
Design, art, print	1.33 (0.69- 2.56)
Construction and interior fitting	0.42 (0.19-0.94)*
Electrical engineering, information technology	0.99 (0.63-1.55)
Metal and machinery	1.32 (0.68-2.54)
Chemistry, physics	0.91 (0.59-1.40)
Planning and construction	0.79 (0.47-1.31)
Sale	1.09 (0.57-2.07)
Economy and administration	0.73 (0.50-1.07)
Traffic, logistics, vehicles	1.14 (0.68-1.92)
Health	1.28 (0.85-1.93)
Education and social affairs	1.10 (0.62-1.93)
Vocational preparation	1.75 (1.00-3.04)*

*Note:* *p < .05, **p < .01. OR = Odds Ratio; CI = Confidence Interval.

### Predictors of risk factor combinations

Predictors of the various risk factor combinations are displayed in Table 6. We found lower prevalence of combined hazardous drinking and tobacco smoking in females compared to males (OR = 0.69, 95% CI = 0.56-0.86, *p* < .01), in persons with a secondary school degree compared to the reference group with no educational degree (OR = 0.56, 95% CI = 0.34-0.91, *p* < .05) and in persons with both parents born outside Switzerland compared to those with none of their parents born outside Switzerland.

**Table 6:** Multiple prediction model of different risk factor combinations (reference group: vocational school students with 0 or 1 risk factor)

**Variable**	**Hazardous drinking & tobacco smoking**	**Hazardous drinking & physical inactivity**	**Tobacco smoking & physical inactivity**	**Hazardous drinking & tobacco smoking & physical inactivity**
	**OR (95% CI)**	**OR (95% CI)**	**OR (95% CI)**	**OR (95% CI)**
Gender: Male (ref.)				
Female	0.69 (0.56-0.86)**	1.77 (1.22-2.58)**	4.67 (2.61-8.35)**	1.93 (1.44-2.59)**
Age: 15-16 years (ref.)				
17-18 years	1.25 (0.95-1.66)	2.15 (1.25-3.72)**	1.47 (0.65-3.31)	1.51 (0.94-2.45)
19-20 years	1.38 (1.00-1.90)	1.92 (1.06-3.48)*	1.88 (0.86-4.10)	2.12 (1.23-3.66)**
21 years or older	1.36 (0.85-2.18)	2.46 (1.12-5.41)*	3.12 (1.17-8.31)*	2.96 (1.54-5.72)**
Educational attainment: No grade (ref.)				
Secondary school	0.56 (0.34-0.91)*	0.46 (0.19-1.10)	0.43 (0.11-1.67)	2.55 (0.64-10.18)
Extended secondary school	0.62 (0.35-1.08)	0.47 (0.18-1.24)	0.49 (0.11-2.18)	2.40 (0.57-10.10)
Technical or high school	0.59 (0.31-1.13)	0.83 (0.26-2.60)	0.09 (0.01-1.12)	2.51 (0.56-11.29)
Immigrant background: No (ref.)				
One parent born outside Switzerland	1.18 (0.93-1.51)	1.18 (0.76-1.81)	1.65 (0.85-3.20)	1.48 (1.00-2.20)
Both parents born outside Switzerland	0.53 (0.41-0.68)**	0.52 (0.34-0.81)**	2.41 (1.47-3.95)**	0.80 (0.58-1.09)

*Note:* *p < .05, **p < .01. OR = Odds Ratio; CI = Confidence Interval.

The combination hazardous drinking and physical inactivity was more prevalent in females (OR = 1.77, 95% CI = 1.22-2.58, *p* < .01), in vocational school students older than 15–16 years, which was the reference group (17–18 years: OR = 2.15, 95% CI = 1.25-3.72, *p* < .01; 19–20 years: OR = 1.92, 95% CI = 1.06-3.48, *p* < .05; 21 years or older: OR = 2.46, 95% CI = 1.12-5.41, *p* < .05), but less prevalent in vocational school students with both parents born outside Switzerland compared to those with none of their parents born outside Switzerland (OR = 0.52, 95% CI = 0.34-0.81, *p* < .01).

The combination tobacco smoking and physical inactivity was more prevalent in females (OR = 4.67, 95% CI = 2.61-8.35, *p* < .01), in vocational school students older than 21 years compared to the reference group of students aged 15–16 years (OR = 3.12, 95% CI = 1.17-8.31, *p* < .05) and in vocational school students with both parents born outside Switzerland compared to those with none of their parents born outside Switzerland (OR = 2.41, 95% CI = 1.47-3.95, *p* < .01).

The combination of all three risk factors was more prevalent in females (OR = 1.93, 95% CI = 1.44-2.59, *p* < .01) and in vocational school students older than 18 years compared to the reference group of those aged 15–16 years (19–20 years: OR = 2.12, 95% CI = 1.23-3.66, *p* < .01; 21 years or older: OR = 2.96, 95% CI = 1.54-5.72, *p* < .01).

## Discussion

The study revealed three main findings: (a) Four out of five vocational school students showed at least one of the investigated health risk factors, hazardous drinking, tobacco smoking, or physical inactivity. (b) Multiple risk factors were ascertained in a significant proportion of vocational school students. Specifically, tobacco smoking and hazardous drinking were coexistent. (c) The study revealed subgroups of vocational school students with a particularly high risk for a single risk factor and for multiple risk factors. These subgroups should be addressed with preventive measures.

The majority of the vocational school students (79.4%) showed at least one of the investigated risk factors, nearly half of the vocational school students (43.6%) had two or more risk factors and 9.6% showed all three health risk factors. Nearly two thirds (62.2%) of the students were drinking hazardously, while about one third of the sample were smoking tobacco (36.9%) or showed low levels of physical activity (28.0%). Hazardous drinking was more prevalent in male, physical inactivity was more prevalent in female vocational school students. The proportion of students with low physical activity and tobacco smoking increased with increasing age.

Due to different methodology and sample selection effects, a comparison of our results with the results of European surveys in secondary schools [5,6] or the aforementioned survey in vocational schools from Germany [10] is possible only to a certain extent. Although the distributions of age and gender were comparable, we found a significantly lower prevalence of daily smokers in our study population than in the German survey at vocational schools (29.4% vs. 56.5%) and a slightly lower prevalence of hazardous drinking (62.2% vs. 78.8%). Considering only vocational school students aged 16 years, we found slightly higher proportions of daily smokers in this sample of vocational school students than in the sample of 16-year-old pupils at secondary schools from the ESPAD-survey [20] (22.4% vs. 19.7%) and much higher proportions of hazardous drinking (57.4% vs. 25.6%). These results indicate that type of school (secondary school vs. vocational school) and formal education, with vocational schools having a higher proportion of students with no or low educational attainment, may contribute to an increased prevalence of health risk factors in vocational school students.

No or only marginal correlations occurred between hazardous drinking and physical inactivity and tobacco smoking and physical inactivity, however we found a moderate correlation between hazardous drinking and tobacco smoking. While 81.3% of the smoking vocational school students were drinking hazardously, this proportion was only 48.5% among non-smoking students. Due to the synergistic negative effects of hazardous drinking and smoking on certain diseases, e.g., oral, pharyngeal and oesophageal cancer [21-23], the group of cigarette smokers with hazardous drinking should be a main target group for initiating preventive measures. Preliminary data support that integrated smoking cessation and binge drinking interventions may enhance smoking cessation and reduce binge drinking [24,25].

Targeted interventions to reduce alcohol consumption should specifically address those subgroups with the highest proportions of hazardous drinking. These subgroups are as follows: males, students aged 17 years or older, students with no educational degree, as well as students from the “design, art, print” and “electrical engineering, information technology” vocational fields. In contrast, we found relatively low proportions of hazardous drinking in students with both parents born outside Switzerland and in those attending vocational preparation. The main countries of origin within the students with immigrant background were Turkey and the former States of Yugoslavia (Kosovo, Bosnia). A substantial part of persons in these countries are Muslims. The religious norms of alcohol prohibition in these population groups might result in a lower prevalence of hazardous drinking. Furthermore, these groups are characterised by having particularly close social networks [26]. These networks might also result in lower socialization with other people (e.g. from Switzerland), e.g. in bars or clubs.

Targeted interventions to reduce tobacco smoking should be specifically addressed to vocational school students aged 19 years and older, to students with one parent born outside Switzerland and to students from the “gastronomy, food” and “beauty, sports” vocational fields. In our study, all students from the latter vocational field were hairdressers. Therefore, we can specify this suggestion to students of this profession.

While hazardous drinking is more prevalent in male students, physical inactivity is significantly more prevalent in females than males. Similar to hazardous drinking and smoking, the proportion of vocational school students with low physical inactivity also increases with increasing age. These gender- and age-specific results concerning physical activity are in line with data from a representative survey on physical activity and exercise in children and adolescents from Switzerland [27]. Vocational groups with the lowest proportions of persons meeting the physical activity recommendations are students in vocational preparation and hairdressers.

While the combination of hazardous drinking and tobacco smoking was higher in males, the other risk factor combinations were observed particularly among females. Furthermore, multiple risk factors were generally more prevalent in students aged 17 years and older compared to those aged 15–16 years. Therefore, targeted interventions addressing drinking and smoking should be specifically addressed to male students aged 17 years and older, interventions addressing drinking and inactivity, smoking and inactivity and interventions addressing all three risk factors should be particularly addressed to female students aged 17 years and older.

One limitation of this study is that the data were not collected from a representative survey of all vocational school students in Switzerland. Therefore, the results could not be generalized for Swiss vocational school students. However, the distributions concerning age groups and gender are quite similar to the national statistics of vocational school students in Switzerland [9]. Another limitation of the study is that the survey did not include students from all possible vocational fields. Additionally, the number of students in certain vocational fields was quite small and did not allow us to detect small to medium effects.

This is the first study examining hazardous drinking, tobacco smoking and physical inactivity in a large sample of vocational school students. Furthermore, it is one of the rare studies which systematically considers the occupational category and immigrant background of the participants. Since nearly half of the vocational school students showed two or more health risk factors, this study underlines the importance of multiple behaviour interventions in this population group. Although first conceptual frameworks for interventions addressing more than one risk factor exist, there is a lack of empirical evidence regarding their acceptance and efficacy [28].

Only a few studies have examined the acceptance and efficacy of interventions that support smoking cessation or that decrease hazardous drinking among vocational school students [29-32]. These studies showed a high acceptance of text messaging based interventions [30,32] or a single group session intervention [31], however, the results concerning their efficacy are preliminary [30,32] or mixed [31]. Brief or low-threshold interventions, such as Internet or text messaging programs as well as brief motivational counselling, could be incorporated within regular school lessons and are well suited to reach a high proportion of vocational school students. For example, Internet based social norms interventions, that were originally developed for college or university students, are also promising for the reduction of hazardous drinking in vocational school students. However, it should be noted that the efficacy of tailored messages depends on the individual’s ability and motivation to process information. The Elaboration Likelihood Model [33] posits that the degree to which individuals are motivated and able to process a persuasive message (Need for Cognition, NFC) determines the attention dedicated to the central points of a message. Individuals with high NFC are motivated to seek information actively and to think about the arguments presented to them. Individuals with low NFC pay more attention to the source of the arguments (e.g., celebrities, credible authorities, experts), the ease with which they can be processed (e.g., presented pictorially versus verbally), and the number of arguments presented, in order to process information. Although NFC is thought to reflect a cognitive motivation rather than an intellectual ability, it is positively correlated with educational level [34]. Taking into account individual NFC could be crucial for improving the outcomes of health behaviour interventions [35-37] for vocational school students.

## Conclusions

One or multiple risk factors were ascertained in a significant proportion of vocational school students. Hazardous drinking was more prevalent in male, physical inactivity was more prevalent in female vocational school students. The proportion of students with low physical activity and tobacco smoking increased with increasing age. Specifically, tobacco smoking and hazardous drinking were coexistent. The study underlines the need for preventive measures in specific subpopulations of adolescents and young adults with lower educational level.

## Competing interests

The authors declare that they have no competing interests.

## Authors’ contributions

SH, CM, and UJ were responsible for the study design. SH and MS were responsible for data collection. SH and CSG were responsible for the data analyses and interpretation. All authors read and approved the final manuscript.

## Pre-publication history

The pre-publication history for this paper can be accessed here:

http://www.biomedcentral.com/1471-2458/13/475/prepub
